# The Theoretical Highest Frame Rate of Silicon Image Sensors

**DOI:** 10.3390/s17030483

**Published:** 2017-02-28

**Authors:** Takeharu Goji Etoh, Anh Quang Nguyen, Yoshinari Kamakura, Kazuhiro Shimonomura, Thi Yen Le, Nobuya Mori

**Affiliations:** 1 Graduate School of Engineering, Osaka University, 2-1 Yamada-Oka, Suita, Osaka 565-0871, Japan; kamakura@si.eei.eng.osaka-u.ac.jp (Y.K.); lethiyen@si.eei.eng.osaka-u.ac.jp (T.Y.L.); nobuya.mori@eei.eng.osaka-u.ac.jp (N.M.); 2 Graduate School of Science and Engineering, Ritsumeikan University, 1-1-1 Noji-Higashi, Kusatsu, Shiga 525-8577, Japan; gr0220pp@ed.ritsumei.ac.jp (A.Q.N.); skazu@fc.ritsumei.ac.jp (K.S.)

**Keywords:** high speed, image sensor, frame rate, temporal resolution limit, BSI

## Abstract

The frame rate of the digital high-speed video camera was 2000 frames per second (fps) in 1989, and has been exponentially increasing. A simulation study showed that a silicon image sensor made with a 130 nm process technology can achieve about 10^10^ fps. The frame rate seems to approach the upper bound. Rayleigh proposed an expression on the theoretical spatial resolution limit when the resolution of lenses approached the limit. In this paper, the temporal resolution limit of silicon image sensors was theoretically analyzed. It is revealed that the limit is mainly governed by *mixing* of charges with different travel times caused by the distribution of penetration depth of light. The derived expression of the limit is extremely simple, yet accurate. For example, the limit for green light of 550 nm incident to silicon image sensors at 300 K is 11.1 picoseconds. Therefore, the theoretical highest frame rate is 90.1 Gfps (about 10^11^ fps).

## 1. Evolution of High-Speed Imaging Devices

In this section, evolution of high-speed imaging devices is summarized, which suggests the importance of searching for the ultimate high-speed of silicon image sensors.

In this paper, a video camera is defined as a camera capable of capturing 100 or more consecutive images. Then, an image can be replayed at 10 frames per second (fps) for more than 10 s. The time constant of the fundamental saccade motion of human eyeballs is 200 ms or less [1]. Therefore, image frames replayed at 10 fps look sufficiently smooth. This criterion was discussed in 1994 [2] and in 2003 [3] and widely accepted in the design of burst image sensors for video cameras [4,5,6,7,8,9,10,11,12]. Cameras and other imaging devices capable of capturing less than 100 consecutive images are referred to as multi-framing cameras or devices [13,14,15,16,17,18,19,20,21,22,23].

The first commercially available digital high-speed video camera was Kodak Ektapro EM, first marketed in 1989 in the USA. The frame rate was 2000 fps for a pixel count of 240 × 192 pixels. Etoh developed a faster one in 1991 [4]. The frame rate was 4500 fps for 256 × 256 pixels to 40,500 fps for 64 × 64 pixels. The camera was marketed as Kodak Ektapro HS4540 and Photron FASTCAM (the first generation).

**Figure 1:**
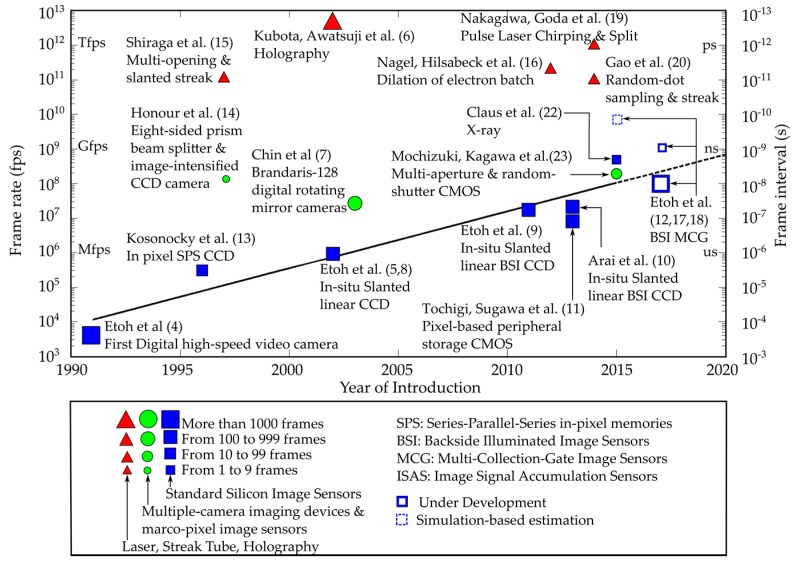
Evolution of high-speed imaging devices.

Since then, the frame rates of digital high-speed cameras have rapidly increased, as shown with blue squares and green circles in Figure 1 [4,5,8,9,10,11,12,13,17,18]. The blue squares indicate the frame rates achieved by standard high-speed cameras with one silicon image sensor. The green circles indicate the frame rates of cameras with a set of multiple cameras [7,14] and a camera with an image sensor with macropixels, each consisting of multiple pixels [23]. The red triangles indicate the frame rates recorded with imaging devices supported by various technologies other than high-speed image sensors [6,15,16,19,20]. The size of each plot represents the frame counts. The squares drawn with a blue solid line and a dashed line indicate, respectively, the frame rates of silicon image sensors under process and under design based on simulations.

Cameras with silicon image sensors are compact, sensitive, and user-friendly. Various useful built-in functions can be installed in the sensor. Therefore, the performance is being rapidly improved. For example, the frame rate has exponentially increased (linearly increased in a semilogarithmic scale in Figure 1) from only about 10^4^ fps in the mid-1990s. Test chips of high-speed image sensors for a video camera and a multi-framing camera respectively operating at 10^8^ fps and 10^9^ fps are under process [12]. For X-ray imaging, image sensors capable of capturing 2 or 4 frames at the frame interval of 2 ns were developed in the image sensor team of Sandia National Laboratories [22]. A simulation study predicted that the shortest frame interval, less than 200 ps (0.5 × 10^10^ fps), can be achieved by a silicon image sensor with a 130 nm process technology both for the sensor structure and the driving circuit for charge detection [21]. On the other hand, the frame rates of other continuous imaging devices mostly stay between 10^11^ fps and 10^12^ fps. The highest frame rate of the cameras with silicon image sensors has come closer to the frame rate achievable only by other high-speed imaging devices in the past.

There is no plot below the trend line indicating the frame rates of the silicon image sensors. Once a silicon image sensor achieves a frame rate, the user-friendliness has eliminated other high-speed imaging devices operating at the frame rate similar to or less than the frame rate of the silicon image sensor. For example, when the digital high-speed video camera of 4500 fps was developed (1991), high-speed film cameras had already achieved a frame rate much higher than 10,000 fps with high-resolution color images. Still, they completely disappeared before 2000. Exploring the ultimate potential of silicon image sensors is essential to forecast the future of the field of high-speed imaging.

The spatial resolution limit was discussed by Abbe, Rayleigh, and others, when the resolution of lenses approached the limit [24]. The frame rate of silicon image sensors seems to approach the upper bound. Research relating to the highest possible frame rate of silicon image sensors has just begun [21,25,26]. It is time to search for the temporal resolution limit.

The highest frame rate of an image senor is limited by either the performance of the photoelectron generation and the transport processes in the photoelectron conversion layer, or that of the detection circuit. The former is the subject of this paper. Fortunately, the temporal resolution limit due to the generation and the transport processes was theoretically derived as described in the following sections.

## 2. Assumptions 

### 2.1. Sensor Structure for Photo-Conversion

Assume the following conditions:

(1) The structure of the image sensor is backside-illuminated (BSI).
(2) One incident photon generates one single charge, if the thickness of the sensor is infinite.
(3) Incident photons are perpendicular to the backside surface.
(4) The camera is equipped with a single image sensor.
(5) All pixels are exposed for the same duration to capture one frame.
(6) Each pixel is equipped with one charge detection circuit on the front side.
(7) Signal charges are electrons.

For visible light, these conditions will be satisfied by most cameras in the very near future, except the third one. The expression of the temporal resolution limit derived in Section 3.1 can be easily generalized to light with distributed incident angles, as explained in Section 4.1.

### 2.2. Target Wavelengths

Blue, green, and red rays with the wavelengths of 450 nm, 550 nm, and 650 nm are selected as to be representative of visible light. Table 1 shows their fundamental properties relating to the present analysis.

### 2.3. Signal Detected by Silicon Image Sensor

A photon of visible light incident to the backside of an image sensor generates an electron–hole pair. Theoretically, an electromagnetic signal is generated due to photoelectron conversion in the backside layer and propagates at the speed of an electromagnetic wave in the silicon crystal to the front side. Turchetta [25] and Mutoh [26] discussed the temporal resolution from this aspect. However, the signal generated at the photoelectron conversion of visible light is too weak to detect with existing detection circuits. The duration of a photoelectron conversion process is much shorter than a travel time of an electron from the backside to the front side. Therefore, the temporal resolution of image sensors for visible light is governed by motions of charges. The mobility of electrons is higher than that of holes. Hereafter, the temporal resolution is analyzed based on motions of electrons detected at the front side of the sensor.

### 2.4. Variation of Travel Time Due to Diffusion and Mixing

In natural phenomena, dispersion is caused by an integrated effect of diffusion due to random motions and mixing due to other macromotions, where dispersion is defined as the total spreading of the distribution. In most cases, mixing enhances dispersion much more than diffusion. In a pipe flow, for example, mixing of slow-speed water near the pipe wall and high-speed water near the center is the major cause of spreading of contaminants in the flow direction.

**Figure 2:**
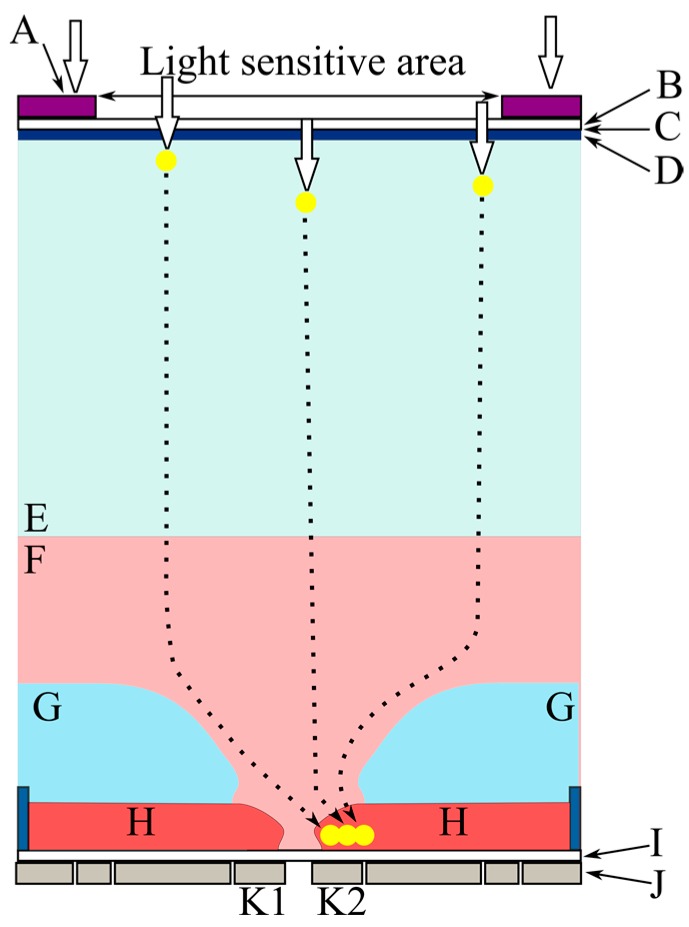
A cross-section structure of a pixel of a backside-illuminated (BSI) image sensor and trajectories of signal electrons. A: light shield; B: oxide; C: interface between the backside oxide layer and the silicon layer; D: backside hole accumulation layer; E: p^−^ layer, F: n^−^ layer; G: p-well; H: memory and circuit areas; I: oxide; J: electrodes and wires; K: collection gates.

**Figure 3:**
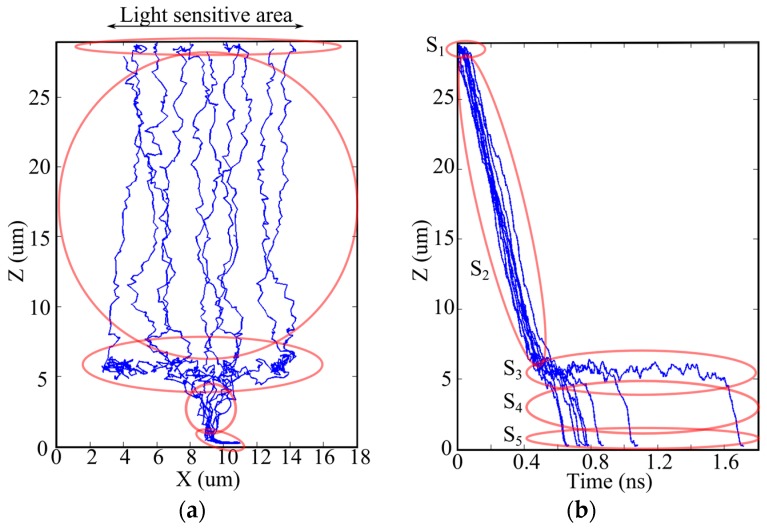
Monte Carlo simulation of trajectories of signal electrons and segments of the trajectories. (**a**) Trajectories; (**b**) travel time versus travel distance from the collection gate. S1: Motion in the backside hole accumulation layer; S2: Motion in the depleted bulk silicon layer; S3: Motion over the p-well; S4: Motion in a p-well hole; S5: Motion to a collection gate.

Figure 2 shows an example cross-section of a pixel of a backside-illuminated image sensor [17,18,21,28]. The thickness of the chip is about 30 µm to prevent a part of incident light remaining after absorption in the silicon layer from direct intrusion to circuits on the front side. A high bias voltage is applied to the backside to deplete the silicon bulk layer. Figure 3 shows an example of Monte Carlo simulations of the trajectories of signal electrons. The motions of the electrons in layers of each pixel are categorized as follows [21]:

1. In a very thin and very high p^+^ layer near the backside (a hole accumulation layer), S1: the layer is filled with holes to eliminate dark current from the backside interface; a nearly isotropic random motion of electrons causes large diffusion.
2. In a bulk depleted layer, S2: drift of electrons is dominant over the random motion; however, the generation sites spread in the vertical direction due to distribution of the penetration depth of light, which causes distribution of the travel time of the generated signal electrons; mixing of electrons with different travel times results in dispersion in addition to the diffusion due to the random motion.
3. In a layer over a p-type umbrella (a p-well) covering the front-side circuits, S3: the p-well is created to prevent direct intrusion of signal electrons to the circuits; the field is inclined and weak, causing a horizontal drift component with relatively large diffusion; distribution of the horizontal travel distance of a signal electron from the distributed generation site to the center causes mixing of electrons; the mixing with relatively large diffusion results in large dispersion in time.
4. In a hole of the p-well around the center of the pixel, S4: signal electrons move vertically; the dispersion resulting from the random motion associated with the vertical drift is not significant.
5. Under the p-well, S5: the motion of electrons is dependent on the circuit structure around the center on the front side.

The hole accumulation layer can be made much thinner than the penetration depth of visible light with an advanced process technology. Then, the whole incident light penetrates the layer and generates electrons in the deeper layer. Therefore, the dispersion in the layer can be neglected in the near future.

**Figure 4:**
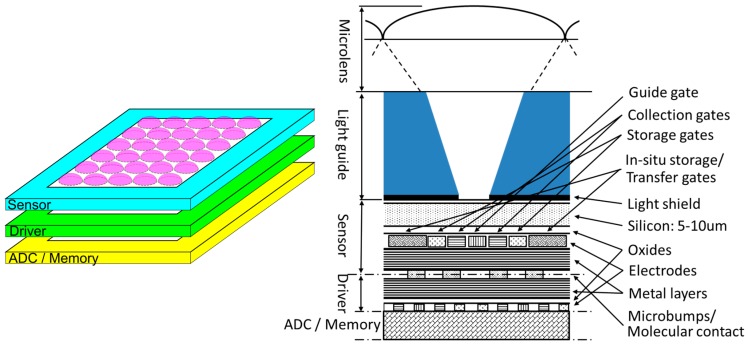
A pixel of a 3D-stacked backside-illuminated multi-collection-gate (BSI-MCG) image sensor with an on-pixel microlens and/or a light guide [21].

The dispersion due to the p-type umbrella can be reduced by design efforts. Figure 4 [21] shows an example. An on-pixel microlens and/or a light guide to focus incident light to the center of each pixel effectively reduces the horizontal mixing. The effect is further enhanced by a light shield with a hole covering the backside.

On the other hand, the mixing due to the distribution of the penetration depth together with the diffusion due to the random motion cannot be avoided with any design effort. Consequently, these two causes are employed to theoretically derive the temporal resolution limit.

### 2.5. Measure of Temporal Resolution

**Figure 5:**
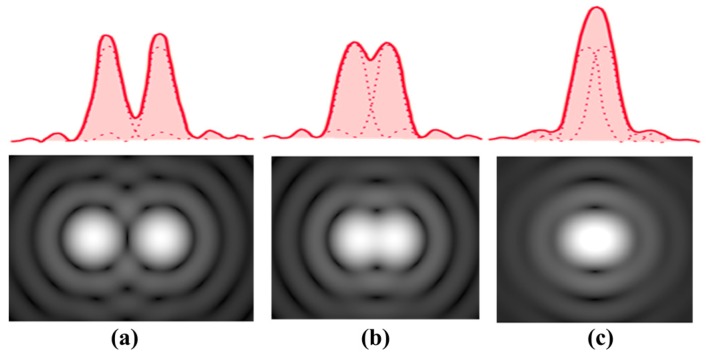
Airy diffraction patterns generated by light from two points passing through a circular aperture. (**a**) Points far apart; (**b**) Rayleigh criterion; (**c**) points closer than Rayleigh criterion [29,30].

The Rayleigh criterion is commonly used as a standard measure of the spatial resolution of optical systems. The criterion is expressed as the distance from the center of the primary Airy disc to the first dark circle. Exactly speaking, the definition does not satisfy the real separation limit, as is well known. The intensity distribution of the superposed two primary Airy discs, according to the Rayleigh criterion, has a dip of 26% at the center as shown in Figure 5 [29,30]. Dowe’s empirical resolution limit prior to Rayleigh’s proposal is closer to the no-dip condition, with only a 6% dip.

The temporal resolution of imaging devices lowers due to dispersion of arrival times of the signals. Therefore, the standard deviation is the primal measure of the resolution. Different measures are employed for the specific problems, such as a standard imaging and time-stamping. They are all related to the standard deviation. To keep consistency with the measure for the spatial resolution, the no-dip condition expressed by the standard deviation is employed in the present analysis. In the spatial resolution analysis of optical systems, a spot image spreads by diffraction; the shape of the intensity distribution is the primary Airy disc. In the temporal resolution analysis presented later, the travel time spreads by dispersion; the shape of the distribution is Gaussian, except for the distribution observed very close to the generation site.

**Figure 6:**
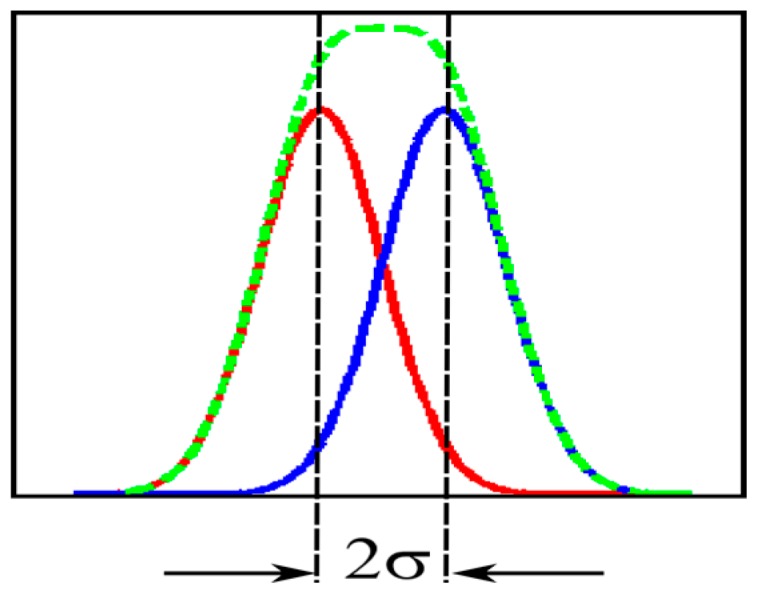
Separation limit (no-dip condition) for two superposed Gaussian distributions; σ: standard deviation.

Assume that two infinitesimal light spots in time and space arrive at the same spot on the backside of the sensor at different times with the interval ∆τ. A batch of signal electrons generated by each light spot travels to the front side, spreading due to diffusion and mixing. The temporal distribution of the dispersion approaches the Gaussian distribution when the travel distance from the backside increases. During the travel, the difference in the average travel time between two batches of electrons is kept at ∆τ. The no-dip condition for two superposed Gaussian distributions is strictly ∆τ = 2σ, as shown in Figure 6, where σ is the standard deviation of one Gaussian distribution. Therefore, the value 2σ is employed as the measure of the temporal resolution [21].

### 2.6. Monte Carlo Simulation Model

A Monte Carlo simulation model developed by Kunikiyo et al. [31] is employed. The model is based on calculation of a precise band structure of an electron in a silicon crystal. The model automatically includes simulation of phenomena appearing in a higher field, such as impact ionization generating secondary electrons, energy allocation of the impacting electron to the secondary electrons and a phonon. Parameters such as the drift velocity *v* and the diffusion coefficient D can be calculated through the simulation. However, the model does not include processes such as a successive impact ionization for a much higher field, recombination of generated electrons and holes, and feedback of the space-field effect of generated electrons and holes.

### 2.7. Drift Velocity and Diffusion Coefficient

**Figure 7:**
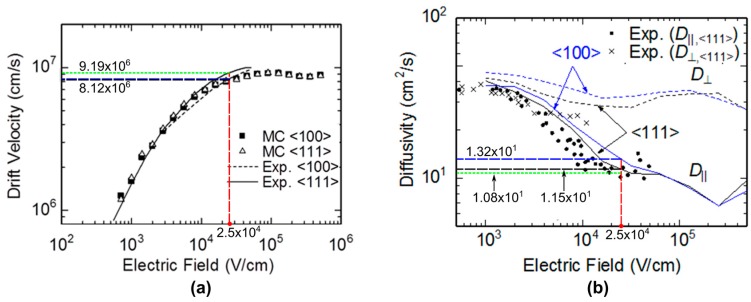
(**a**) Drift velocity with respect to electric field at 300 K [31,32]; (**b**) diffusivity with respect to electric field at 300 K [31,33].

Impurity dopants decrease the drift velocity *v* and increase the diffusion coefficient D, which enhances dispersion. Therefore, an intrinsic silicon layer is assumed to derive the theoretical temporal resolution limit. Figure 7a,b respectively show the dependency of *v* and D on the field *E* for the intrinsic silicon at 300 K. The values of *v* and D calculated by the advanced Monte Carlo simulations are compared with the experimental data [30,31,32].

For the drift velocity, the results of the Monte Carlo simulation agree well with the experimental data. The drift velocity increases proportionally to the field in a normal operation range of fields, which are less than 1.0 × 10^4^ V/cm, and approaches a constant value at the field around 2.5 × 10^4^ V/cm due to frequent scattering events by higher-energy electrons colliding with the silicon lattice. Hereafter, the value of the field for the saturated drift velocity, 2.5 × 10^4^ V/cm, is referred to as *a critical field*.

The Monte Carlo simulation model can separately provide the drift velocities and the diffusion coefficients in the directions parallel to and perpendicular to the travel direction and for different crystal orientations. The simulated diffusion coefficients are slightly higher than the experimental data. Another important discrepancy between the simulated and experimental data appear at the field higher than the critical field. The vertical diffusion coefficient D parallel to the travel direction reduces along the field until the critical field. The experimental data seem to take a minimum at the critical field. On the other hand, the Monte Carlo simulation results (curves) further decrease for the higher field. The discrepancy may be due to the insufficient expression for fundamental processes for the very high field in the Monte Carlo simulation model explained in Section 2.6. Naturally, the cascade generation process of the secondary electrons which appear in a very high field may increase the diffusion coefficient again. 

The drift velocity and the diffusion coefficient for the crystal orientation <111> are higher and lower, respectively, than those for <100>. Therefore, the values for <111> are employed to calculate the values of the temporal resolution limit.

## 3. Expression of Standard Deviation of Travel Time 

### 3.1. Derivation

**Figure 8:**
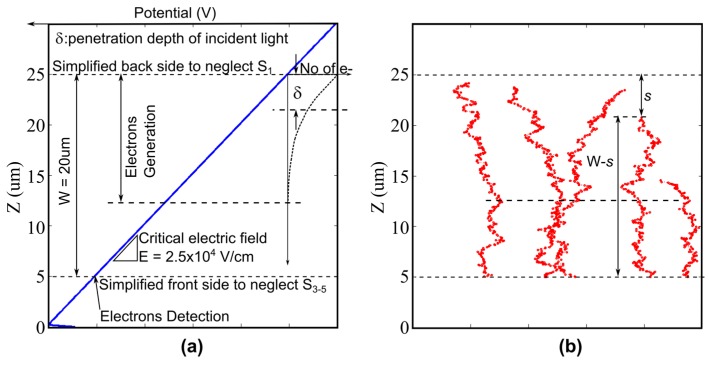
A cross-section structure for derivation of the standard deviation σ of the travel time for W = 20 µm. (**a**) The model for the analysis; (**b**) example trajectories of electrons.

The structure of a BSI image sensor is simplified to include the factors unavoidable and essential for the analysis of the temporal resolution limit, as shown in Figure 8. The model includes the distribution of the penetration depth of photons and the random motion of signal electrons. A constant field is created by a backside bias voltage.

The travel distance of an electron generated at the depth *s* from the backside to the front side is (W − *s*), where W is the thickness of the chip. The average travel time *t_r_* is (W − *s*)/*v*. The probability density function f(*s*) of the penetration depth *s* is as follows:

(1) f(s)=(1 /δ) exp (−s / δ)

where *δ* is the average penetration depth.

The zeroth to the second moments of the average travel time around the origin are:

(2) $$p=\int_{0}^{W}f(s)\;\mathrm{ds}=1-\exp(-W/\delta )$$

(3) $$E(t_{r})=\int_{0}^{W}\{(W-\;s)\;/\;v\}(f(s)\;/\;p)\;\mathrm{ds}=(W-\;\delta p)/(v\;p)$$

(4) $$\begin{array}{ll} E({t_{r}}^{2}) & =\int_{0}^{W}{\left\{(W-s)/v\right\}}^{2}(f(s)/p)\;\mathrm{ds}\\ & =(W^{2}-\;2W\delta \;+\;2\delta^{2})\;/\;v^{2}+\frac{W^{2}\;-\;2W\delta }{v^{2}p}\exp\;(-\;W\;/\;\delta ) \end{array}$$

Then, the variance *σ_m_*^2^ due to the mixing effect of electrons penetrating to different depths is:

(5) $${\sigma_{m}}^{2}=E({t_{r}}^{2})\;-\;{\left\{E(t_{r})\right\}}^{2}$$

After some cumbersome manipulation of equations, the expression for *σ_m_*^2^ was fortunately reduced to a very simple form:

(6) $${\sigma_{m}}^{2}=\frac{W^{2}}{p^{2}v^{2}}[-\exp(-W/\delta )\;+\;\frac{\delta^{2}}{W^{2}}p^{2}]=-\frac{\delta^{2}{W\prime }^{2}}{p^{2}v^{2}}\exp(-W\prime )+\frac{\delta^{2}}{v^{2}}$$

where W’ = W/*δ*.

Random motion of signal electrons is superposed on the drift motion. The solution of the drift and diffusion equation shows that, when the travel time increases, the travel time distribution approaches the Gaussian distribution [34]. The average travel time is *t_r_* and the variance is 2D/*v*^2^. Therefore, the variance is proportional to *t_r_* multiplied by 2D/*v*^2^. The average travel time *t_r_* distributes due to the distribution of the penetration depth of light. Therefore, the variance σ_d_^2^ due to the diffusion effect is given by integration of the variance conditional to the average travel time weighted by the distribution of the penetration depth. Then,

(7) $${\sigma_{d}}^{2}=\frac{2D}{v^{2}}E(t_{r})=\frac{2D}{v^{2}}\int_{0}^{W}\{(W-s)\;/\;v\}(f(s)\;/\;p)\;\mathrm{ds}=\;\frac{\left(W\prime -p\right)}{p}\frac{2D}{v^{2}}\frac{\delta }{v}$$

The mixing due to the distribution of the penetration depth and the diffusion conditioned by the average travel time are independent processes. Therefore, the total variance *σ_s_*^2^ is the sum of *σ_m_*^2^ and *σ_d_*^2^.

(8) $${\sigma_{s}}^{2}={\sigma_{m}}^{2}+{\sigma_{d}}^{2}$$

Then, the temporal resolution limit ∆τ is

(9) $$\Delta \tau =2\sigma_{s}$$

Equation (9) is generally applicable to photo-charge conversion layers, which satisfy the conditions stated in Section 2.1. The value is calculated by specifying the values of the parameters included in the expression: the drift velocity, the diffusion coefficient, and the thickness of the layer. 

Sensitivity is crucial in ultrahigh-speed imaging. The quantum efficiency seriously decreases for the thickness less than the average penetration depth. The temporal resolution limit becomes larger for an increase of the thickness as shown in Equations (6)–(9). Therefore, the thickness can be safely assumed to be equal to the average penetration depth (i.e., W’ = W/*δ* = 1). 

Then, *p* = 1 − *e*^−1^ = 0.6321, and the temporal resolution limit is:

(10) $$\Delta \tau =2\sqrt{0.079\frac{\delta^{2}}{v^{2}}+0.582\frac{2D}{v^{2}}\frac{\delta }{v}}$$

Later, Monte Carlo simulations for silicon image sensors show that *σ_m_* >> *σ_d_*. Then, the diffusion effect *σ_d_* (the second term) is neglected. The value of the drift velocity *v* for the experimental data in Figure 7a is 9.19 × 10^−2^ µm/ps. Then, the expression of the temporal resolution limit is reduced to an extremely simple form:

(11) $$\Delta \tau =0.562\frac{\delta }{v}=6.12\;\delta$$

where the units of ∆τ and *δ* are, respectively, ps and µm.

It is proved in Section 3.2 that the accuracy of Equation (11) is sufficiently high.

### 3.2. Comparison with Monte Carlo Simulation

Equation (9) is compared with results of Monte Carlo simulations for an intrinsic silicon layer. The diffusion coefficient estimated by the Monte Carlo simulations is slightly higher than the experimental data, as shown in Figure 7b. Therefore, in comparing the derived expression of the temporal resolution limit with the result of the Monte Carlo simulations, the values of *v* and D estimated through the Monte Carlo simulations are used. To finally estimate the temporal resolution limit later, the values estimated from the experimental data are substituted into the derived expression (9).

For the critical field 2.5 × 10^4^ V/cm at 300 K, the drift velocity and the diffusion coefficient for the crystal orientation <111> are, respectively, 8.12 × 10^6^ cm/s and 11.5 cm^2^/s.

**Figure 9:**
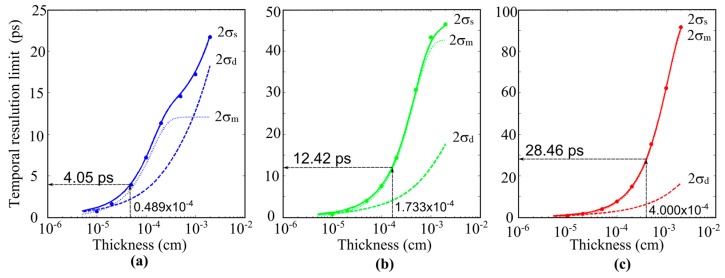
Proof of the derived expression of the temporal resolution limit. (Solid lines: the temporal resolution limit 2*σ_s_*, Equation (9); dotted lines: the mixing effect 2*σ_m_*, Equation (6); dashed lines: the diffusion effect 2*σ_d_*, Equation (7); dots: Monte Carlo simulations.) (Intrinsic silicon <111>; 300 K; electric field: 2.5 × 10^4^ V/cm; wavelength: (**a**) 450 nm; (**b**) 550 nm; (**c**) 650 nm.)

Example results are shown in Figure 9 for blue, green, and red light of 450 nm, 550 nm, and 650 nm. The solid lines and the dots respectively indicate the temporal resolution 2σ_s_ calculated from Equation (9) and by the Monte Carlo simulations. They agree almost perfectly for a thickness larger than 1 µm (10^−4^ cm). The agreement is confirmed for the field range of 0.5 × 10^4^ V/cm to 2.5 × 10^4^ V/cm, though the results are not shown.

The dashed lines and the dotted lines further show the mixing effect 2*σ_m_* and the pure diffusion effect 2*σ_d_*. They show the following characteristics:

(1) For a thickness more than 1 µm, the agreement is almost perfect. For a thickness less than 0.5 µm, the theoretical value is slightly larger than the result of the Monte Carlo simulation. The reason is the large skew of the distribution of the travel time observed near the generation site.
(2) The temporal resolution of blue light of 450 nm shows a strange curve. The reason is that the curve expresses a compound effect of two comparable and completely different effects due to the pure diffusion and the mixing. For a thickness less than 3 µm, the mixing effect is dominant; for a thickness larger than 3 µm, the mixing effect saturates and the diffusion effect becomes dominant.
(3) For green and red light, the mixing effect due to the distribution of the penetration depth is dominant, and the diffusion effect is practically negligible.

The average penetration depth *δ* is shown in Table 1. For example, for the green light, *δ* = 1.733 µm. Then, the temporal resolution limit ∆τ is 12.4 ps from the figure or Equation (10). Similarly, for blue and red light, ∆τ is 4.05 ps and 28.5 ps, respectively.

An approximate value of the temporal resolution limit ∆τ is calculated with Equation (11) as 12.0 ps, which is sufficiently close to 12.4 ps obtained from Equation (10).

When the experimental data shown in Figure 4 and Figure 5 for the critical field are employed, instead of the values calculated by Monte Carlo simulations, the drift velocity is 9.19 × 10^6^ cm/s and the diffusion coefficient is 10.8 cm^2^/s. Then, the temporal resolution limit for green light is 11.1 ps for Equation (10). Therefore, the upper-bound frame rate is 90.1 Gfps.

If we use Equation (11), ∆τ = 10.7 ps (error: 4%), in spite of the extremely simple expression. For the blue and the red light, the errors are 14% and negligible.

### 3.3. Summary of Logic

The temporal resolution limit of photo-charge conversion layers is theoretically derived. It is expressed with an extremely simple expression. Since the derivation is complicated with several logical steps, the flow is summarized as explained below ((i)–(vii)). As an example, the temporal resolution limit of silicon image sensors is estimated from the expression and the values of the electronic parameters of silicon crystals ((viii)–(v)).

(i) The resolution is limited by the distribution of travel times of signal charges.
(ii) There are two unavoidable factors affecting the distribution: (a) *mixing* of signal electrons with different travel distances due to the distributed penetration depths of photons; (b) *pure diffusion* due to random motions of electrons.
(iii) The expressions of variances σ_m_^2^ and σ_d_^2^ of the travel time caused by the mixing and the diffusion are separately derived and added, since they are independent processes.
(iv) The no-dip condition for two superposed Gaussian distributions is employed as the temporal separation limit of batches of electrons generated by two photon groups each instantly arriving at the layer with a time difference ∆τ, where the no-dip condition is 2σ, and σ is the standard deviation of one Gaussian distribution (Equation (9)).
(v) The derived expression includes drift of the velocity *v*, the diffusion coefficient D, and the average penetration depth *δ*. The temporal resolution limit 2σ is calculated by substituting the values of *v*, D, and *δ* into Equation (9).
(vi) Equation (9) is verified by the comparison with the results of the Monte Carlo simulations for the values of *v* and D for the critical field.
(vii) The thickness W is assumed to be equal to the average penetration depth *δ* to prevent the sensitivity from decreasing for *δ* < W, and the temporal resolution from increasing for *δ* > W. Therefore, W’ = W/*δ* = 1, which provides Equation (10).
(viii) For silicon layers, the temporal resolution 2σ is minimized by the values of *v* and D at the critical field, 2.5 × 10^4^ V/cm.
(ix) For example, for green light of 550 nm, the temporal resolution limit is 11.1 ps for Equation (10), which is equivalent to 90.1 Gfps.
(x) Later, Monte Carlo simulations show *σ_m_* >> *σ_d_* for the silicon image sensors for ranges of W in practical applications. Therefore, if the diffusion effect *σ_d_* is neglected and the value of *v* for the critical field is inserted, the expression of the temporal resolution limit is finally reduced to an extremely simple expression in Equation (11). The error to the value calculated from Equation (10) is only 4% for green light.

The derivation is based on some assumptions. For example, the arrival time distribution is expressed as the flux distribution in time passing through a detection plane, which differs from Gaussian distribution when the travel time is short. Though it is possible to formulate the expression of the temporal resolution limit by using the precise expression of the temporal flux distribution, the result is expressed as an implicit equation and requires numerical calculations to see the characteristics of the solution. The derived expression in this paper is explicit and theoretically sound, from which the fundamental characteristics are clearly observed.

## 4. Generalization of Present Theory 

### 4.1. Distributed Incident Angles of Light

The average penetration depth *δ* in Equation (9) is replaced with a standard deviation *σ_v_* of vertical components of penetration depths of visible rays with distributed incident angles. The accuracy of the expression will be sufficiently high in practice.

### 4.2. Cooled Sensor

Cooling is a very important technology for scientific image sensors to decrease dark current noise and decrease cross-talks due to diffusion of signal charges. It is expected that cooling the sensor may improve the temporal resolution. However, Equation (11) implies that the temperature dependency is not significant. At lower temperatures, both the drift velocity *v* and the average penetration depth *δ* increase together and compensate each other’s effects. For example, at about 170 K, the values of *v* and *δ* increase by about 40% [32] and 60% [35], respectively, compared with those at 300 K. Then, the temporal resolution limit even increases by 14% (1.6/1.4 = 1.14).

### 4.3. X-ray

The analytical framework presented in this paper can be modified for analyses of the temporal resolution for more generalized cases. For example, for a soft X-ray, Landau distribution of the number of charges suddenly generated by a single photon and Gaussian distribution of the initial spread of the batch of the charges should be incorporated into the present analysis. Since these processes are independent of the mixing and the diffusion processes, the variance of the arrival time due to these effects may be simply added to Equation (8).

In this case, the arrival time is generally measured as the time when the number of charges generated by a single photon and detected by a detection circuit reaches a specified value. The time is also distributed due to the generation and the transportation processes in the photo-charge conversion layer and temporal fluctuations of the detection circuit elements. The latter effect on the temporal resolution has been analyzed in the field. When the detection circuit is improved, the former one will become the target of the research.

### 4.4. Macropixel Image Sensor

An image sensor with macropixels each consisting of several neighboring pixels is referred to as *a macropixel image sensor*. If each pixel in a macropixel receives photons at different times and the temporal resolution of the detection circuit of each pixel is infinitesimal, the temporal resolution limit of the macropixel image sensor is much less than the temporal resolution limit expressed by Equation (9). 

Assume a macropixel image sensor with macropixels each consisting of two pixels. Batches of charges generated by two photon groups incident to the two elemental pixels are not mixed and separately detected, and the average travel times are independently calculated. The variance of the average travel time is proportional to the variance σ_s_^2^ expressed by Equation (8) divided by the number of charges *N*. The statistical significance of the difference between the average travel times of two batches is tested with the t-test. Therefore, the temporal resolution limit may be defined by the difference corresponding to a given significance level. For *N* > 30, the t-distribution can be approximated by a Gaussian distribution. Therefore, if the significance level is given, the temporal resolution limit is easily calculated.

If the target event is reproducible, the temporal resolution can be improved by repeating the imaging. If the number of repetitions is M, the variance is further reduced to 1/M. The result correctly holds for a macropixel image sensor with more elemental pixels, and for imaging devices consisting of more than two cameras.

The number of consecutive images of a macropixel image sensor is the number of the elemental pixels in each macropixel. A disadvantage of the macropixel is reduced sensitivity, inversely proportional to the number of consecutive frames. Loss of sensitivity is crucial in ultrahigh-speed imaging.

If different color filters are attached to *K* elemental pixels of each macropixel, and lasers with wavelengths corresponding to the color filters are applied at a very short interval for illumination, *K* consecutive images are captured at the short frame interval. The wavelength of a laser can be swept at a very high speed. Chirping can be used to sweep the wavelength at an extremely high speed. This kind of ultrahigh-speed imaging technologies is sometimes called *hyperspectral methods*. The authors call them *time-spectrum conversion methods*. The most advanced one of such methods developed by Goda et al. [19].

With *time-space conversion methods*, image information of different instants is recorded at different positions. The positions are changed at a very high speed. The position-changing methods include a rotating mirror for shooting images on a circular surface (a rotating mirror camera) [7], deflection electrodes to streak electron beams (a streak tube) [14], even a flying light beam [6], and so on.

Random sampling in space or time with advanced signal process technologies are also applied in ultrahigh-speed imaging to distinguish image information captured at different instances.

Most of these technologies utilize image sensors as the final recording devices of image information. The discussion on the temporal resolution of the macropixel image sensor can be directly applied for analysis of the temporal resolution of the image sensors for these ultrahigh-speed imaging technologies. However, the temporal resolution of macropixel image sensors and independently operated multiple standard image sensors in these applications is usually much shorter than the temporal resolutions of other elements of the devices. Therefore, the overall temporal resolution is limited by a critical element of each imaging device or system other than the image sensors.

## 5. Conclusions

The temporal resolution limit of silicon image sensors is theoretically derived. The limit is mainly governed by *mixing* of charges with different travel times caused by the distribution of penetration depth of light. The final expression is ∆τ = 6.12 *δ*, which may be unbelievably simple, but sufficiently accurate. Now, the target is clear. It is time to give it a try to break it.

## Figures and Tables

**Table 1:** Fundamental properties of light for a silicon crystal [27].

Wavelength (nm)	Photon Energy (eV)	Absorption Coefficient (cm^−1^)	Penetration Depth (µm)
450	2.753	2.05 × 10^4^	0.489
550	2.255	5.77 × 10^4^	1.733
650	1.907	2.05 × 10^3^	4.000
